# Is this ‘complicated’ opioid withdrawal?

**DOI:** 10.4103/0019-5545.31604

**Published:** 2006

**Authors:** S.R. Parkar, R Seethalakshmi, S Adarkar, S Kharawala

**Affiliations:** * Professor and Head Department of Psychiatry and Drug Deaddiction Centre, K.E.M. Hospital, Mumbai, Maharashtra, India; ** Resident Medical Officer Department of Psychiatry and Drug Deaddiction Centre, K.E.M. Hospital, Mumbai, Maharashtra, India; *** Lecturer Department of Psychiatry and Drug Deaddiction Centre, K.E.M. Hospital, Mumbai, Maharashtra, India; **** Resident Medical Officer Department of Psychiatry and Drug Deaddiction Centre, K.E.M. Hospital, Mumbai, Maharashtra, India

**Keywords:** Opioid, withdrawal syndrome, convulsions, delirium

## Abstract

Seven patients with opioid dependence admitted in the de-addiction centre for detoxification developed convulsions and delirium during the withdrawal phase. After ruling out all other possible causes of these complications, opioid withdrawal seemed to emerge as the most likely explanation. The unpredictability of the course of opioid dependence and withdrawal needs to be considered when treating patients with opioid dependence.

## INTRODUCTION

Traditionally, opioid withdrawal is characterized by severe muscle cramps, profuse diarrhoea, abdominal cramps, rhinorrhoea, lacrimation, fever, yawning, piloerection, hypertension, pupillary dilatation, tachycardia and temperature dysregulation (described as *cold turkey*). Patients seldom have other complications unless they have co-morbid medical illnesses. This report describes a series of 7 inpatients with opioid dependence who developed uncommon clinical complications during the withdrawal phase.

## THE CASE

Seven patients with opioid dependence admitted for detoxification in our inpatient facility developed uncommon clinical complications during the withdrawal phase. Four of these patients developed delirium; 3 developed generalized tonic-clonic convulsions, of whom 2 patients further developed altered sensorium. The onset of these complications varied from 3 to 7 days since the last consumption. The patients' ages ranged between 20 and 38 years and the duration of consumption was between 2 and 6 years. The primary mode of consumption was inhalation of vapours formed from opium heated on an aluminum foil; 3 patients also had a history of intravenous use. Urinary opioid levels done by the radioimmunoassay method ranged from 0 to 2000 ng/dl. One of these patients had a history of co-morbid benzodiazepine dependence; the rest denied any history of benzodiazepine abuse or dependence. Laboratory investigations to confirm this could not be carried out. All 7 patients denied history of alcohol use, which was confirmed by urinary tests for alcohol. The caretakers of the patients confirmed the patients' histories regarding co-morbid substance dependence. None of the 7 patients had any prior history of head injury, convulsions or any other disease. Routine blood parameters were within the normal ranges. Medications used for the management of withdrawal prior to the development of complications included either substitution with dextro-propoxyphene or benzodiazepines or haloperidol for behavioural management. CT scan done on a delirious patient and electroencephalography of a patient with convulsions did not reveal any abnormality. All the patients recovered with no clinical evidence of any neurological sequelae. In view of these unreported complications, an attempt was made to obtain the opioid consumed by these patients; however, this proved fruitless. The opioid that all the patients consumed was the street variety, which is generally contaminated.

## DISCUSSION

Complications such as convulsions and delirium are recognized in alcohol withdrawal. However, these have not been described as a feature of opioid withdrawal. In these 7 patients, all the likely causes of these complications were ruled out, and opioid withdrawal emerged as a probable cause of these complications. There is, however, no literature on the subject. Neonatal abstinence syndrome (NAS) due to opiate withdrawal in infants of opioid-dependent mothers is known to result in epileptiform activity with seizures that may be generalized and myoclonic.^1^ Precipitation of narcotic withdrawal in rats has been shown to be associated with increased cerebral activity that is largely unnoticed in narcotic withdrawal.^2^ A high incidence of delirium—20% over a period of 1 year—has been reported following rapid opioid detoxification with naltrexone and clonidine in methadone-dependent patients.^3^

Although the majority of opioid withdrawal symptoms are somatic in nature, various neurological conditions have also been described in patients with opioid dependence. Two unusual cases of opioid dependence who developed neurological complications have been detailed by Celius.^4^ One patient developed rhabdomyolysis, bilateral cortical infarctions and convulsions after an intravenous injection of heroin, while the second patient developed an acute disabling cerebellar ataxia after intra-arterial injection of heroin. Rhabdomyolysis presenting as mononeuropathy, plexus lesions^5^ and transverse myelitis^6^ has been reported in heroin addicts. De Gans *et al.* concluded that an allergic or toxic reaction to heroin or adulterants was more likely to be the cause of these complications.^5^ Unusual manifestations of ischaemic lesions in young patients with a history of drug abuse include border zone infarcts in the spinal cord and the cerebral cortex.^7^ Heroin-related spongiform leukoencephalopathy has been described in a patient with history of intravenous use;^8^ epidemiological studies have suggested that the inhalatory use of poisoned heroin vapours (pyrolysate) could be the cause of spongiform encephalopathy in some patients.^9^ However, none of these conditions were observed in any of our patients; the causative role of these being thus doubtful. Co-morbidity is the rule rather than exception in patients with substance dependence. Though all our patients denied any other co-morbid substance dependence, except one who had benzodiazepine dependence, the reliability of an addict's history is often questionable. The caretakers are also usually oblivious to the nature of the substances taken.^10^ In view of the lack of literature, it is most likely that the complications manifested in these patients were due to concurrent use of another substance such as alcohol or benzodioazepines. Another possibility is the presence of a contaminant, which we were unfortunately unable to confirm. Nonetheless, the potentially life-threatening nature of these complications warrants that patients with primarily opioid dependence be carefully monitored. ‘What the mind doth not know, the eyes do not see.’
